# Biogeographic origin and phylogenetic relationships of *Mepraia* (Hemiptera, Reduviidae) on islands of northern Chile

**DOI:** 10.1371/journal.pone.0234056

**Published:** 2020-06-11

**Authors:** Ricardo Campos-Soto, Gabriel Díaz-Campusano, Ninette Rives-Blanchard, Franco Cianferoni, Fernando Torres-Pérez

**Affiliations:** Instituto de Biología, Facultad de Ciencias, Pontificia Universidad Católica de Valparaíso, Valparaíso, Chile; Washington State University, UNITED STATES

## Abstract

Chagas disease is one of the main zoonoses mediated by vectors in America. The etiological agent is the protozoan *Trypanosoma cruzi*, transmitted mainly by hematophagous insects of the subfamily Triatominae. *Mepraia* species are triatomines endemic to Chile that play an important role in *T*. *cruzi* transmission in the wild cycle and are potential vectors for humans. In addition to the continental distribution, populations of *Mepraia* genus have been reported inhabiting islands of northern Chile. The presence of individuals of *Mepraia* in insular areas might be explained through passive dispersion by marine birds or by vicariance of an ancestral widespread population. To clarify the biogeographic origin and phylogenetic relationships of island individuals of *Mepraia*, mitochondrial *COI* and *cyt b* genes were sequenced in individuals from island and continental areas. Gene sequences were used to estimate phylogenetic relationships, divergence dates and migration rates between insular and continental populations. The dates of divergence estimates are congruent with sea level and tectonic changes that originated the islands during Pleistocene. Migration rates suggest symmetric historical island-continent gene flow. We suggest that the origin of island triatomines can be explained by both vicariance and dispersion. Phylogenetic relationships show that individuals from Santa María Island and the continent clustered in a clade different from those previously reported, indicating a new lineage of *Mepraia* genu*s*. This study will contribute to understand the origin of the *T*. *cruzi* infection in coastal islands of northern Chile.

## Introduction

Phylogeographic and biogeographic historical processes of hosts, vectors and pathogens are crucial to understand the current epidemiological status of zoonotic diseases and provide critical information for control management [1,2]. Chagas disease is one of the main zoonotic diseases mediated by vectors in America. The etiologic agent is the protozoan *Trypanosoma cruzi*, which is transmitted principally by hematophagous hemipterans of the subfamily Triatominae. *Mepraia* is a genus [3] of Triatominae endemic to arid and semiarid regions of Chile; it plays an important role in *T*. *cruzi* transmission in the wild, and its species are potential vectors for humans [4,5]. Three species are currently included in the genus *Mepraia*: *M*. *gajardoi* is distributed from 18°30´S to 24°34´ S exclusively in coastal areas [6–9]; *M*. *parapatrica* was also reported inhabiting coastal areas from 24°34´ S to 27° S [6,7,9]; *M*. *spinolai* inhabits coastal areas from 28° S to 30° 44´ S and interior valleys from 26° 30´ S to 34°20´ S. *M*. *parapatrica* is distributed in the coastal desert in an area intermediate between the ranges of *M*. *spinolai* and *M*. *gajardoi* [6,7,9].

Island populations of *Mepraia* genus were reported inhabiting in Pan de Azúcar Island at 1.8 km from the continent (26° 9'S) in the Atacama Region [10]; these individuals were characterized as *M*. *parapatrica* using morphological approaches [6]. Insular individuals of *Mepraia* were also reported on Santa María Island at 1.9 km from the continent (23°28' S) in the Antofagasta Region [11] an area located between *M*. *gajardoi* and *M*. *parapatrica* distribution [7]. Interestingly, the presence of kissing bugs infected with *T*. *cruzi* were reported both on Pan de Azúcar and Santa María Islands [11]. The biogeographic origin of the island triatomines is unknown. There are two hypotheses that may contribute to explain the biogeographic origin of these island triatomines. The first hypothesis is related to the origin of insular triatomines by dispersion. Sagua et al.[10] suggested that passive transport by marine birds might explain the occurrence of individuals of *Mepraia* on Pan de Azúcar Island. An alternative hypothesis explains that the populations on islands resulted from vicariant processes splitting the continent and islands due to sea level changes [6]. Supporting the latter hypothesis, important sea level fluctuations and tectonic changes during the Pleistocene were reported in the areas where Santa María and Pan de Azúcar Islands occur [12–16]. Therefore, both dispersal and vicariance processes could have played a role in the occurrence of individuals of *Mepraia* on islands of northern Chile. The vicariance and dispersion hypotheses can be tested by estimating the divergence time, haplotypic differentiation and historical migration rates between island and continental populations. According with the dispersal hypothesis, recent genetic divergence, shared haplotypes, low genetic differences and high migration rates between continent and island populations are expected. For the vicariance hypothesis, estimated dates of divergence congruent with the Pleistocene epoch, different haplotypes and significant genetic differences between islands and continental populations are expected [17–19].

The phylogenetic relationships that individuals of *Mepraia* found in those islands compared to the three current species (clades) is unknown. We used phylogenetic analyses, estimations of the time to the most recent common ancestor (TMRCA), and estimation of migration rates between island and continent populations to: i) determine the biogeographic processes leading to the occurrence of triatomines on islands from northern Chile, and ii) determine their phylogenetic relationships with all reported *Mepraia* species. We expect that the results of this study will also contribute to understand the origin of the *T*. *cruzi* infection in coastal islands of northern Chile.

## Materials and methods

### Areas of triatomine collection

**Fig 1:**
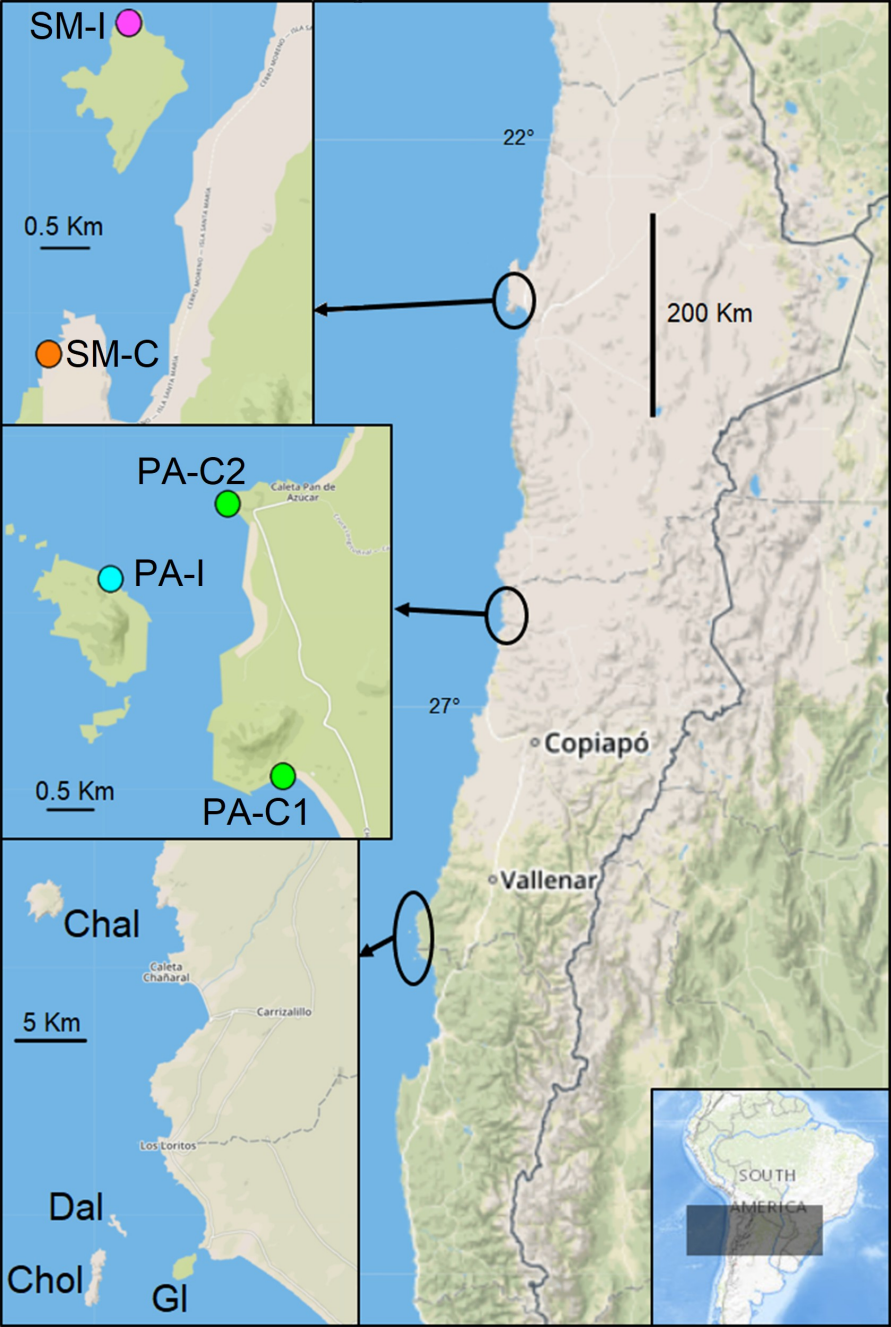
Map of the studied islands. Sampling localities of triatomines. SM-I: Santa María Island, SM-C: Santa María continent, PA-I: Pan de Azúcar Island, PA-C: Pan de Azúcar continent. Map was extracted from WHISPers [21] and modified by illustrations purposes.

Individuals of *Mepraia* were collected during the summer (2017 to 2019) in four coastal zones of northern Chile: Santa María Island (SM-I, N = 16); Santa María continent in front of the island (SM-C N = 17); Pan de Azúcar Island (PA-I N = 35) and Pan de Azúcar continent (PA-C1 N = 34, PA-C2 N = 8). The last two sampling localities are included within the Pan de Azúcar National Park. Two areas of the interior of the valleys were also included: Inca de Oro (Inca N = 9) and San Felipe (SF N = 4, Table 1). Five additional localities were explored (Chañaral Island, Damas Island, Choros Island, Gaviota Island and Punta Choros), but triatomines were not detected (Table 1 and Fig 1). Insects were passively collected by trained people as described in Campos-Soto et al [20] and actively by lifting stones in rock piles and nests. Samples of *Triatoma eratyrusiformis* and *Triatoma breyeri* were used as outgroup for phylogenetic analyses.

**Table 1:** Geographical coordinates of collection localities of individuals of *Mepraia* from insular and continent zones.

Locality	Latitude/longitude	N	*h*	Species
Santa María Island (SM-I)	23°25'51"S/70°36'31"W	16	2	Undetermined
Santa María Continent (SM-C)	23°28'3"S/70°37'2"W	17	2	Undetermined
Pan de Azúcar Island (PA-I)	26° 9'6"S/ 70°41'7"W	35	5	*M*. *parapatrica*
Pan de Azúcar Continent (PA-C1)	26°10'27"S/70°39'58"W	34	1	*M*. *parapatrica*
Pan de Azúcar Continent (PA-C2)	26° 8'34"S/ 70°40'10"W	8	1	*M*. *parapatrica*
Inca de oro (Inca)	26°48'15"S/69°57'14"W	11	3	*M*. *spinolai*
San Felipe (SF)	32°51'24"S/70°29'13"W	4	1	*M*. *spinolai*
Chañaral Island	29° 2'17"S/ 71°34'8"W	0	0	--
Damas Island	29°14'4"S/ 71°31'43"W	0	0	--
Choros Island	29°16'43"S/71°32'17"W	0	0	--
Gaviota Island	29°15'9"S/71°28'20"W	0	0	--
Punta Choros	29°15'5"S/71°27'33"W	0	0	--

N: Number of individuals; *h*: number of haplotypes; 0: individuals not found. Species determination according to [6], [7] and [8].

### Ethics statement

Fields research were authorized by the Corporacion Nacional Forestal (CONAF) from Atacama Region, Chile (permit N° 049/2017) and CONAF from Coquimbo Region (permit N° 22/2019) Chile. The research project that includes this study was approved by the Bioethic Committee of the Pontificia Universidad Católica de Valparaíso (permit # BIOEPUCV-A98b-2017).

### DNA extraction, mitochondrial amplification and sequencing

DNA was extracted from legs of bugs using the DNeasy® Blood & Tissue kit. A gene segment of 636 base pairs (bp) for cytochrome oxidase subunit I (*COI*) and a segment of 682 bp for cytochrome b (*cyt b*) were amplified by the Polymerase Chain Reaction (PCR) using the polymerase SapphireAmp® fast PCR Master Mix. We used the primers described in Folmer et al. (1994) [22] for the *COI* gene, and the primers described in Monteiro et al. (2003) [23] for the *cyt b* gene. The same conditions were used to amplify the *cyt b* and *COI* genes: initial denaturation at 94°C for 3 min, 30 cycles of 1 min at 94°C for denaturation, 45°C for 1 min of annealing, and 72°C for 1 min of extension, followed by a final extension of 10 min. Amplification was verified by 1% agarose gel electrophoresis. Sequencing of both strands was performed by Macrogen Inc. (South Korea) using the same PCR primers. Sequences were edited to obtain consensus sequences using Bioedit 7.0.4.1 [24] and aligned using Clustal W [25] as implemented in Bioedit. After alignment, sites that showed nucleotide substitutions were re-examined by visual inspection of each individual’s raw chromatogram (see S1 Data). *COI* and *cyt b* gene sequences were deposited in GenBank with accession numbers MN117859-MN117888 (S1 Data). Additional GenBank sequences of both *COI* and *cyt b* genes were also included (accession numbers available in S1 Data[7]). Outgroup samples were sequenced for the *COI* gene, while *cyt b* gene sequences were extracted from GenBank. Sequences of both genes of each individual were manually concatenated (S1 Data), and the haplotype data file was obtained using DnaSP6 [26]. The best-fitting model of nucleotide substitution (HKY + G for *COI* and TN93 for *cyt b*) was selected with the Bayesian information criterion (BIC) using Smart Model Selection [27] in the ATGC bioinformatics platform [28].

### Estimates of Time to the Most Recent Common Ancestor (TMRCA)

Haplotype sequences were partitioned by gene and codon position and linked using BEAUti, implemented in BEAST v.2.5.2 [29]. TMRCA were estimated through the Bayesian Markov Chain Monte Carlo (BMCMC) method available in BEAST v.2.5.2 [29]. The coalescent Bayesian skyline was used as tree prior, and run using both strict and uncorrelated lognormal relaxed molecular clocks. The best molecular clock was estimated using model comparison by AICM [30] available in Tracer v.1.6 [31]. The resulting best estimate was the strict molecular clock. We assumed a fixed mean substitution rate of 0.016 sub/site/my estimated for *COI* gene in hemipterans [32], and 0.023 sub/site/my for *cyt b* gene in Triatominae [23] Two separate MCMC runs of 5 × 10^8^ generations, sampling every 50000 generations, were run using the BEAST program. Mixing and convergence on the basis of the effective sample size (ESS) values of each run were verified in Tracer v.1.6 and combined in LogCombiner; only ESSs >200 were accepted. We discarded 10% burn-in, and the statistical uncertainty was depicted in values of the 95% Highest Probability Density (HPD) with mean node heights selected in TreeAnnotator. A consensus tree was calculated with TreeAnnotator and visualized in FigTree v1.4.3. The Median Joining method [33] implemented in PopART software [34] was used to visualize haplotype distribution of island and continent samples.

### Migration rate estimates analysis

Migration rates were estimated by Migrate-n v.3.6.11 software [35]. The software estimates migration rates and effective population sizes between two populations using genetic data, under either maximum likelihood or Bayesian frameworks [36]. These analyses were done with a concatenated matrix using all the individual sequences from each population (Table 1). We ran two independent analyses, first comparing SM-I and SM-C, and then PA-I and PA-C populations. Analyses were run under a full model using Bayesian inference strategy. The parameter M_ij_ defines the proportion of genes of population *j* that comes from population *i* per generation and with size *Ne*. M_i_ is calculated as the immigration rate per generation (*m*_*i*_) divided by mutation rate per site per generation (μ), consequently M_i_ = *m*/μ. The parameter θ_i_ is defined as θ_i_ = *xNe*μ where *x* is the inheritance parameter equivalent to 1 for mtDNA data [36]. Effective number of migrants (*Nm*) was estimated as *Nm =* M_ij_ θ_i_. M_ij_ and θ_i_ parameters were based on *F*_*ST*_ calculations. Four Metropolis-Coupled Chains (5 million visited genealogies, 1,000 recorded steps) were run, with a burn-in of 10,000 iterations. We used an adaptive heating scheme with 4 chains with temperatures set by default (swapping interval  =  1) to increase the efficiency of the MCMC search. A heatmap was constructed with the migration rates estimated from Migrate-n using R package ggplot2 [37]. Genetic differences between populations were estimated with *F*_*ST*_ (pairwise difference) and its significance with a test of 1000 permutations using Arlequin v.3.5.2.2 [38].

## Results

### Phylogeographic relationship and estimates of TMRCA

The sample sizes collected for each locality and their geographic locations are detailed in Table 1 and Fig 1. We obtained a matrix of 125 concatenated *COI* (508 bp) and *cyt b* (514 bp) gene sequences that produced 14 haplotypes. After the inclusion of GenBank sequences, the complete matrix (1022pb) resulted in 47 haplotypes. In the BMCMC analyses, all ESS values were >200.

**Fig 2:**
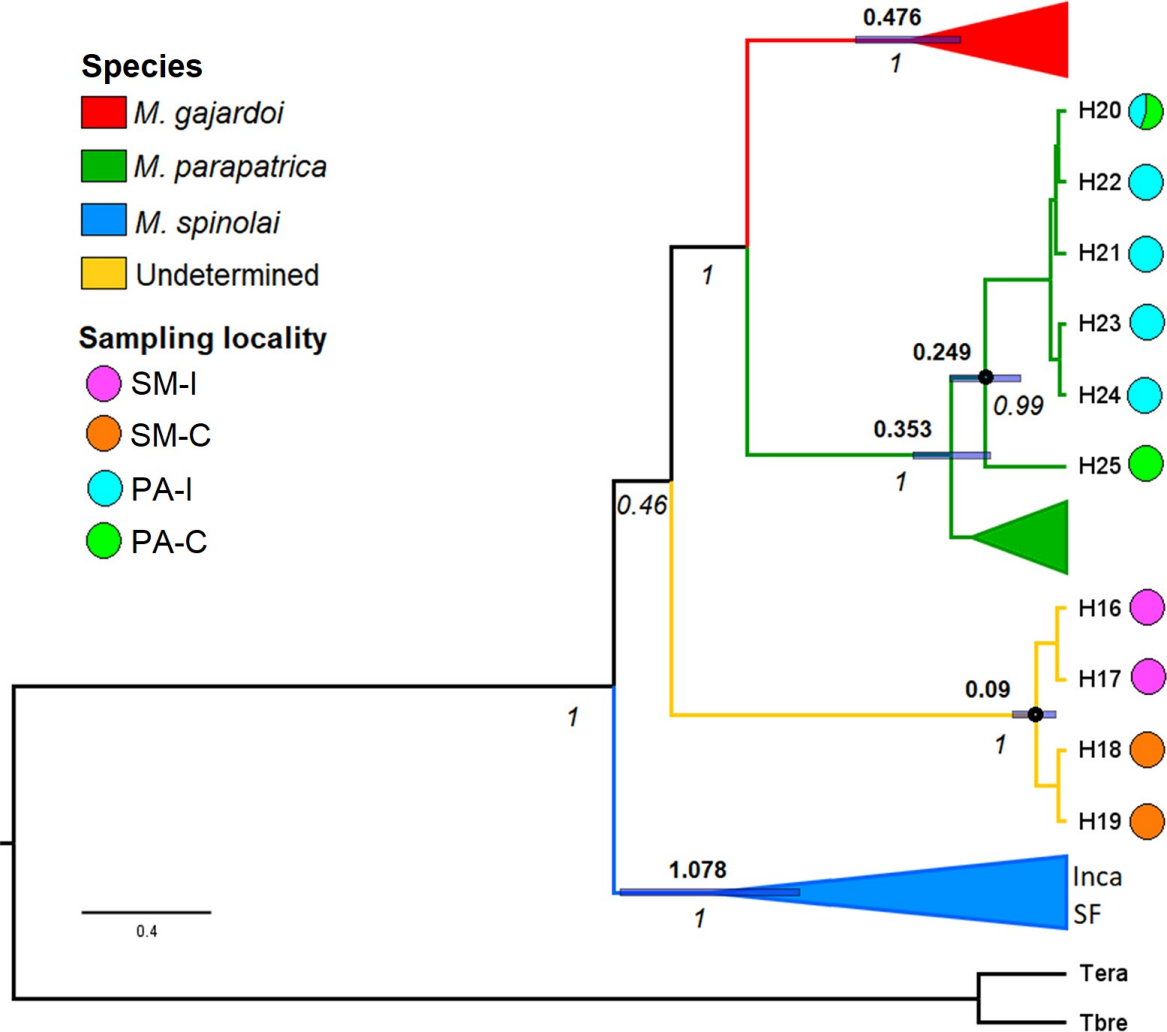
Phylogenetic relationships and estimated date of divergence. Coalescence Bayesian Skyline consensus tree obtained from BEAST with haplotype data using mitochondrial *COI* and *cyt b* gene sequences. Clades collapsed are haplotypes extracted from GenBank except those from Inca de Oro (Inca) and San Felipe (SF). Numbers above the branches are age of most recent common ancestor (in millions of years ago), and blue bars represent 95% highest posterior densities. Numbers below the branches are the Bayesian posterior probabilities. Colors are congruent with localities shown in Fig 1.

**Fig 3:**
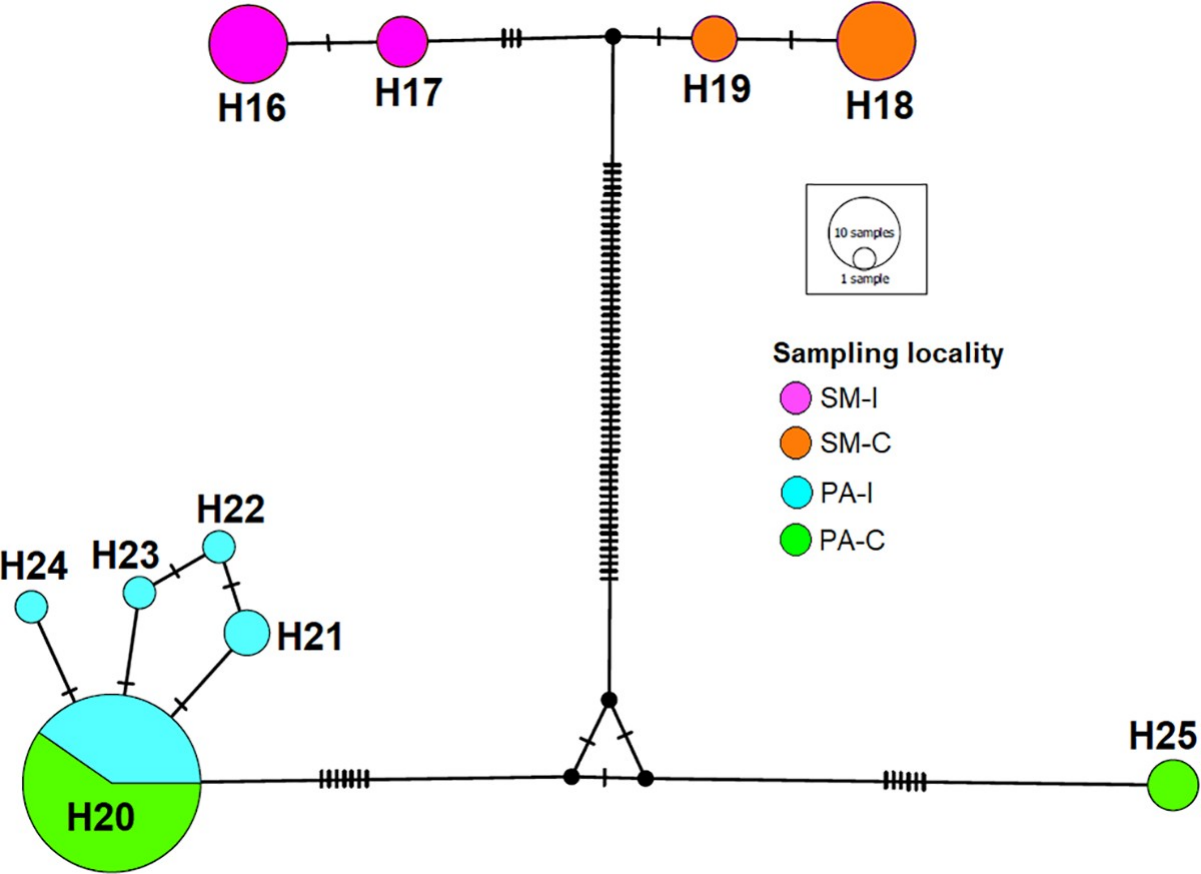
Phylogeographic network. Unrooted phylogeographic network using *COI* and *cyt b* gene sequences with haplotypes depicted according to colors and sampled localities in Fig 1. SM-I: Santa María Island, SM-C: Santa María continent, PA-I: Pan de Azúcar Island, PA-C: Pan de Azúcar continent. Size of circles represents the number of individuals per haplotype.

Samples from Pan de Azúcar Island (PA-I) and Pan de Azúcar continent (PA-C) were included in the *M*. *parapatrica* clade, while those from Santa María Island (SM-I) and Santa María continent (SM-C) were included in a separate clade different from any previously reported species (yellow branch in Fig 2). Inca and SF samples were included within the *M*. *spinolai* clade. The estimated TMRCA for the *M gajardoi* clade was 0.476 million years ago (Mya) (95% HPD = 0.324–0.647), for the *M*. *parapatrica* was 0.353 Mya (95% HPD = 0.233–0.471) and for the *M*. *spinolai* was 1.078 Mya (95% HPD = 0.809–1.361), The estimated TMRCA between SM-I and SM-C haplotypes was 0.09 Mya (95% HPD = 0.032–0.162), and the TMRCA between PA-I and PA-C was estimated at 0.249 Mya (95% HPD = 0.139–0.355, Fig 2). One haplotype is shared between PA-I and PA-C localities (Fig 3).

### Migration rate estimates

**Fig 4:**
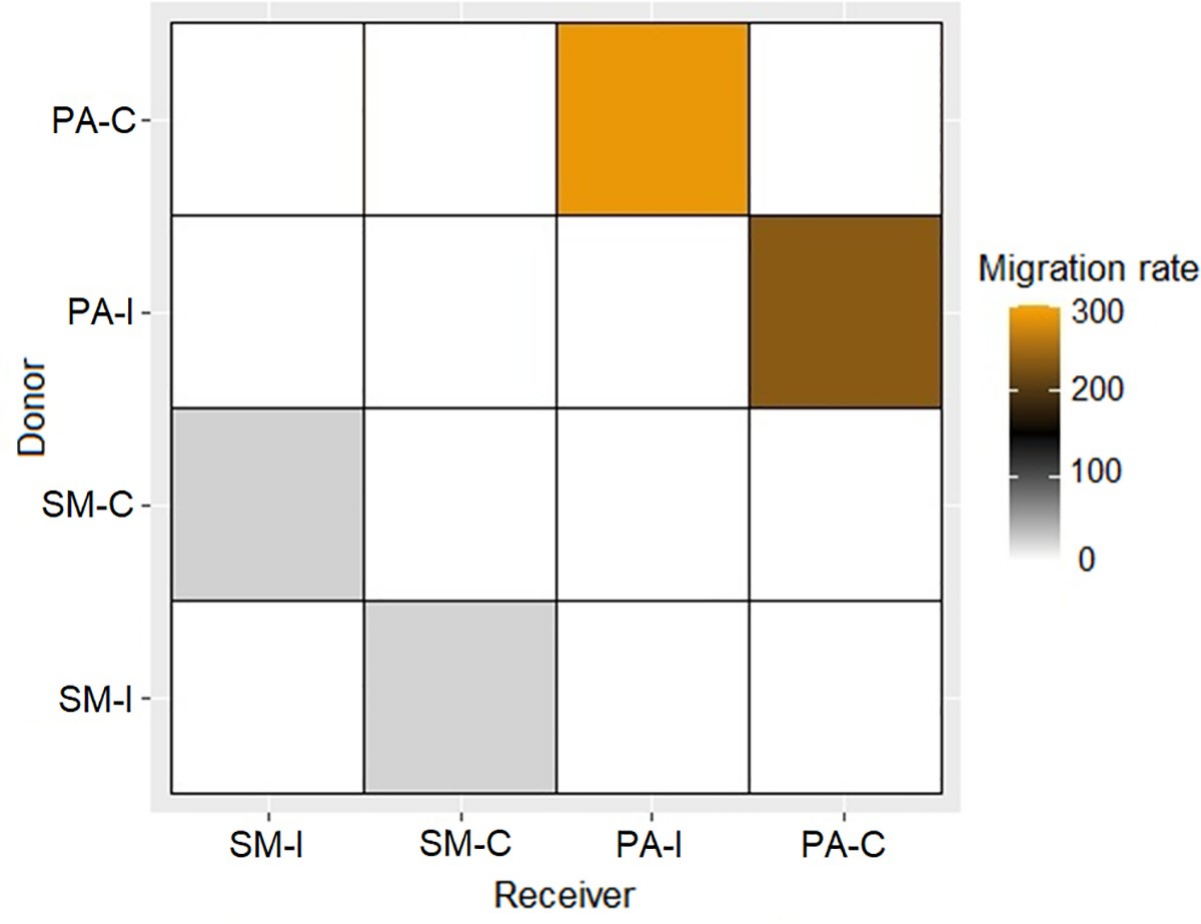
Graphical heat map of migration rates between populations. SM-I: Santa María Island, SM-C: Santa María continent, PA-I: Pan de Azúcar Island, PA-C: Pan de Azúcar continent.

The migrate-n analyses showed good full model performance. The posterior distribution of all parameters resulted in convergence and no warning was recorded during the run. The analyses estimated symmetric gene flow between SM-I and SM-C and between PA-I and PA-C (Table 2, Fig 4). Migration between PA-I and PA-C was particularly strong in both directions, with high levels of migrants per generation (M_PA-I/PA-C_ = 234.9, M_PA-C/PA-I_ = 288.6, Table 2, Fig 4) and effective number of migrants (*Nm*_PA-I/PA-C_ = 16.89, *Nm*_PA-C/PA-I_ = 20.65). Migration between SM-I and SM-C was also detected, although lower compared to PA-I and PA-C (M_SM-I/SM-C_ = 22.9, *Nm*_SM-I/SM-C_ = 1.4, M_SM-C/SM-I_ = 24, *Nm*_SM-C/SM-I_ = 1.48, Table 2, Fig 4). Population comparisons: *F*_*ST*_ was low between PA-I and PA-C (*F*_*ST*_ = 0.091 P<0.016) and high between SM-I and SM-C (*F*_*ST*_ = 0.904 P<0.001).

**Table 2:** Migration rate (M_ij_) values per generation between populations.

	Donor			
Receiver	SM-I	SM-C	PA-I	PA-C
**SM-I**	0.061 (0.05–0.084)	24 (0.1–72.8) 1.48*		
**SM-C**	22.9 (0.1–69.4) 1.4*	0.061 (0.05–0.084)		
**PA-I**			0.072 (0.05–0.096)	288.6 (0.1–825.4) 20.65*
**PA-C**			234.9 (0.1–766) 16.89*	0.072 (0.05–0.096)

Analyses implemented in Migrate-n. M_ij_ defines the proportion of genes of population *j* that comes from population *i* per generation. SM-I: Santa María Island, SM-C: Santa María continent, PA-I: Pan de Azúcar Island, PA-C: Pan de Azúcar continent. Numbers in parenthesis are the 95% confidence interval constructed from minimum and maximum estimates of 0.025 and 0.975 percentiles. Theta values (Θ) for each population are shown on the diagonal. Numbers with * represent the effective number of migrants (*Nm*).

## Discussion

### Biogeographic origin of island triatomines of northern Chile

Processes that determine the origin of populations occurring in insular areas are traditionally explained by dispersal, vicariance, and/or a combination of both. Particularly for zoonotic diseases, understanding the origin and maintenance of the pathogen in its wild cycle has acquired more relevance in the last decades [2,39]. Our study shows that both dispersal and vicariance hypotheses are not exclusive and have played major roles in the biogeographic origin of individuals of *Mepraia* in islands of northern Chile.

Sagua et al.[10] suggested that the occurrence of individuals of *M*. *parapatrica* in Pan de Azúcar Island could be explained through passive transport by marine birds. *Mepraia* species have opportunistic habits, being able to feed on birds both in the continent [40,41] and in the island [10]. Triatomines hidden in cracks may be attracted to birds and make their way between the feathers to feed; they may attach to the feathers and the stylet would pricks the skin. If the stylet remains inserted during feeding, some triatomines would stay attached to their prey. This behavior has been observed under laboratory conditions, when individuals (of the three species of *Mepraia* genus) were feeding on rodents (Campos-Soto, observation).

Pan de Azúcar and Santa María Islands are separated from the continent by about 1.5 km, a distance that marine birds are able to reach without major difficulty. Birds have been suggested to be passive carriers of triatomines and are one of the dispersal mechanisms of triatomines over large areas [42,43]. Alternative dispersal mechanisms of triatomines include people (park rangers and poachers), who sail to the islands carrying insects or eggs in their clothes or backpacks. Vectors and pathogens have been historically transported due to human migration, such as *T*. *infestans* and other triatomines [42–44]. Therefore, if *T*. *cruzi* has infected the transported kissing bug, the likelihood of spreading the pathogen to new geographic areas exists.

Parapatric speciation was proposed as the main mechanism for the appearance of *M*. *parapatrica*, and its occurrence on Pan de Azúcar Island by vicariance [6]. The latter suggests that insular and continental populations were connected and formerly part of an ancestral population that inhabited a wider area. Our molecular analyses showed that the estimations of divergence dates between populations from Pan de Azúcar Island and Pan de Azúcar continent are not recent (0.249 Mya 95% HPD = 0.139–0.355, Fig 2). However, a frequent shared haplotype (H20, see Fig 3), low genetic difference (*F*_*ST*_ = 0.091 P<0.016) and high migration rates (Table 2, Fig 4) suggest that historical dispersal has been a major driver for the occurrence of *M*. *parapatrica* on this island. Despite finding a shared haplotype, our results showed that there are also several derived haplotypes found only in Pan de Azúcar island (Fig 3). This result suggests that both vicariance and dispersal processes have played a major role in the occurrence of triatomines on Pan de Azúcar Island.

We found more recent divergence (0.09 Mya 95% HPD = 0.032–0.162, Fig 2) between Santa María Island and Santa María continent populations compared to that of Pan de Azúcar Island and the continent. Also, absence of shared haplotypes (Fig 3), high genetic differences (*F*_*ST*_ = 0.904 P<0.001) and low migration rates (Table 2, Fig 4) were found between populations of Santa María Island and Santa María continent, suggesting a weak role of dispersal and a probable major role of vicariance.

Geological data shows that the continuous tectonic and sea level changes in marine terrace formation during the Pleistocene-Holocene [12–16] formed both islands, resulting in allopatric splitting of terrestrial populations. Therefore, if enough time has passed to prevent the flow of kissing bug migrants and passive dispersal was not strong, divergent haplotypes between islands and continental populations are expected, and estimations of old divergence dates. According to the International Chronostratigraphic Chart [45], our estimated dates of divergence between Pan de Azúcar Island and Pan de Azúcar continent (0.249 Mya 95% HPD = 0.139–0.355, Fig 2) and between Santa María Island and Santa María continent (0.09 Mya 95% HPD = 0.032–0.162, Fig 2) are in the middle-upper Pleistocene. These ages are congruent with estimated dates (0.03 to 0.7 Mya) of tectonic and sea level changes in marine terraces that impacted the coast in this zone [12–16]. A previous study with scorpions suggested that tectonic changes shaped their current population structure, and that coastal desert species adapted to habitats that have had continuous sea level changes [46].

Intensive sampling in other islands of northern Chile (Damas, Choros, Gaviota and Chañaral) did not result in capture of individuals of *Mepraia* (Table 1, Fig 1). These islands are within the geographic distribution of *M*. *spinolai* [7,8]. *M*. *spinolai* has colonized only some coastal areas (28° S to 30° 44´ S) different from *M*. *gajardoi* and *M*. *parapatrica*, in which the colonization route has been reported mainly from north to south along the coast [6]. We hypothesize that the current absence of individuals of *Mepraia* in those islands may be explained by the absence of this kissing bug when the islands were formed or that ancient allopatric populations were extinguished. Later, the low occurrence of kissing bug populations in continental coastal areas close to those islands (e.g. Punta Choros, Table 1), coupled with the distance from the continent, might prevent their colonization by passive (e.g. bird) transport. Other factors not yet evaluated may also be influencing the absence of kissing bugs in those islands such as food (host) availability.

### Biogeography and phylogeny of *Mepraia* species

The origin and dispersal routes of *Mepraia* genus are still under discussion. *Mepraia* species and *Triatoma eratyrusiformis* would have originated from an ancestral population that diverged due to the uplift of the Andes Range [6,9,47,48]. When Frías [6] described *M*. *parapatrica*, he proposed an origin and latitudinal dispersion of the genus from north to south, suggesting a model of parapatric speciation. *M*. *gajardoi* would be the northernmost and most ancestral species. followed by *M*. *parapatrica* and *M*. *spinolai* as the most recent species. The first molecular systematic study in *Mepraia* genus found that with nuclear markers, the individuals that inhabit in *M*. *parapatrica* areas clustered with the *M*. *spinolai* clade, while with a mitochondrial marker were included in *M*. *gajardoi* clade. This incongruence was explained as a result of mitochondrial introgression due to past hybridization [49], suggesting a possible hybrid origin of *M*. *parapatrica*. A posterior study using two mitochondrial genes found three supported clades congruent with the described species and estimated that *M*. *spinolai* is the most ancestral species of the genus. Consequently, the dispersion and subsequent population differentiation and speciation would have occurred from south to north [7]. Populations from Pan de Azúcar Island and continent were included with significant support in the *M*. *parapatrica* clade (green branch in Fig 2). We expected this result because these populations occur in the range where *M*. *parapatrica* occurs [6,7, 8, 50]. The topology of our phylogenetic tree slightly differs from that previously reported by [7], which can be explained due to the nature of our current analyses that involves adding new samples, new criterion for the phylogeographic inferences, and the uncertainty associated with low-support of bootstrap values of some clades reported in the previous phylogenetic tree of Campos et al. (2013) [7]. Although the Santa María Island and Santa María continental populations are located between the distributions of *M*. *parapatrica* and *M*. *gajardoi*, our results showed that samples from these populations were grouped with high support in a clade different from all previously reported species (yellow branch in Fig 2). However, the phylogenetic relationship of this new lineage remains unclear due to the low support (PP = 0.46, Fig 2) in the phylogenetic tree. Additional molecular markers coupled with morphological information may help to solve this uncertainty.

### Insights to understand *T*. *cruzi* infection in coastal islands of Chile

The *T*. *cruzi* life cycle alternates between triatomines and mammalian host species, while birds and reptiles are refractory to infection [51,52]. It has been reported that individuals of *M*. *parapatriica* feed mainly on seabirds and reptiles in Pan de Azúcar Island [10]; however, hosts have not been studied in Santa María Island due to the recent description of the occurrence of kissing bugs [11]. Strikingly, triatomines infected with *T*. *cruzi* were reported both in Pan de Azúcar and Santa María Islands, raising the question of how the *T*. *cruzi* life cycle is completed with the high abundance of hosts refractory to infection. We speculate that infected triatomines were likely present in the islands since they were formed and historical events of migration carrying infected triatomines from the continent to the islands may have contributed to the current detection of infected kissing bugs. Maintenance of the *T*. *cruzi* life cycle requires mammal hosts, therefore we do not rule out the presence of small mammals inhabiting these islands. Additional studies including captures and surveys of mammals as hosts of *T*. *cruzi* will help to elucidate this pattern.

## Conclusions

In this study we show that the biogeographic processes of vicariance and dispersal may have contributed to the origin of island triatomines in the north of Chile. Also, using phylogenetic inference we identified a new lineage of *Mepraia* that includes haplotypes occurring both in the northern island populations of Chile and the near continental areas. The identification of biogeographic processes leading to the occurrence of kissing bugs on islands and the new lineage of *Mepraia* genus will provide new insights to understand the *T*. *cruzi* life cycle in insular areas of northern Chile.

## Supporting information

S1 Data (DOCX)

## References

[1] Lloyd-Smith JO, George D, Pepin KM, Pitzer VE, Pulliam JRC, Dobson AP, et al Epidemic dynamics at the human-animal interface. Science. 2009;326: 1362–7. 10.1126/science.1177345 19965751 PMC3891603

[2] Pérez-Losada M, Cabezas P, Castro-Nallar E, Crandall K a. Pathogen typing in the genomics era: MLST and the future of molecular epidemiology. Infect Genet Evol. 2013;16: 38–53. 10.1016/j.meegid.2013.01.009 23357583

[3] Mazza S, Gajardo R, Jörg M. *Mepraia* novum genus de Triatominae. *Mepraia spinolai* (Porter) 1933, redescripción del macho y descripción de la hembra. MEPRA Publicación. 1940;44: 3–30.

[4] Campos-Soto R, Ortiz S, Ivan C, Bruneau N, Botto-Mahan C, Solari A. Interactions between *Trypanosoma cruzi* the Chagas disease parasite and naturally infected wild *Mepraia* vectors of Chile. Vector-Borne Zoonotic Dis. 2016;16: 165–171. 10.1089/vbz.2015.1850 26771702

[5] Botto-Mahan C, Sepúlveda M, Vidal M, Acuña-Retamar M, Ortiz S, Solari A. *Trypanosoma cruzi* infection in the sylvatic kissing bug *Mepraia gajardoi* from the Chilean Southern Pacific Ocean coast. Acta Trop. 2008;105: 166–169. 10.1016/j.actatropica.2007.11.003 18177821

[6] Frías D. A new species and karyotype variation in the bordering distribution of *Mepraia spinolai* (Porter) and *Mepraia gajardoi* Frías et al (Hemiptera: Reduviidae: Triatominae) in Chile and its parapatric model of speciation. Neotrop Entomol. 2010;39: 572–583. 10.1590/s1519-566x2010000400017 20877994

[7] Campos R, Torres-Pérez F, Botto-Mahan C, Coronado X, Solari A. High phylogeographic structure in sylvatic vectors of Chagas disease of the genus *Mepraia* (Hemiptera: Reduviidae). Infect Genet Evol. 2013;19: 280–286. 10.1016/j.meegid.2013.04.036 23665465

[8] Frías D, Henry A, Gonzalez C. *Mepraia gajardoi*: a new species of tritominae (Hemiptera: Reduviidae) from Chile and its comparison with *Mepraia spinolai*. Rev Chil Hist Nat. 1998;71: 177–188.

[9] Monteiro FA, Weirauch C, Felix M, Lazoski C, Abad-Franch F. Evolution, Systematics, and Biogeography of the Triatominae, Vectors of Chagas Disease. Advances in Parasitology. 2018 pp. 265–344. 10.1016/bs.apar.2017.12.002 29530308

[10] Sagua H, Araya Rojas J, González Cortes J, Neira Cortes I. *Mepraia spinolai* in the Southeastern Pacific Ocean coast (Chile)—first insular record and feeding pattern on the Pan de Azúcar island. Mem Inst Oswaldo Cruz. 2000;95: 167–170. 10.1590/s0074-02762000000200006 10733734

[11] Rives-Blanchard N, Torres-Pérez F, Ortiz S, Solari A, Campos-Soto R. *Trypanosoma cruzi* over the ocean: Insular zones of Chile with presence of infected vector *Mepraia* species. Acta Trop. 2017;172: 229–231. 10.1016/j.actatropica.2017.05.020 28522273

[12] Pedoja K, Husson L, Johnson ME, Melnick D, Witt C, Pochat S, et al Earth-Science Reviews Coastal staircase sequences reflecting sea-level oscillations and tectonic uplift during the Quaternary and Neogene. Earth Sci Rev. 2014;132: 13–38. 10.1016/j.earscirev.2014.01.007

[13] Binnie A, Dunai TJ, Binnie SA, Victor P, González G, Bolten A. Accelerated late quaternary uplift revealed by 10Be exposure dating of marine terraces, Mejillones Peninsula, northern Chile. Quat Geochronol. 2016;36: 12–27. 10.1016/j.quageo.2016.06.005

[14] Ortlieb L, Zazo C, Goy JL, Hillaire-Marcel C, Ghaleb B, Cournoyer L. Coastal deformation and sea-level changes in the northern Chile subduction area (23 degrees S) during the last 330 ky. Quat Sci Rev. 1996;15: 819–831. 10.1016/S0277-3791(96)00066-2

[15] Ortlieb L, Diaz A, Guzman N. A warm interglacial episode during oxygen isotope stage 11 in northern Chile. Quat Sci Rev. 1996;15: 857–871. 10.1016/S0277-3791(96)00062-5

[16] Quezada J, González G, Dunai T, Jensen A, Juez-Larré J. Alzamiento litoral Pleistoceno del norte de Chile: edades 21Ne de la terraza costera más alta del área deCaldera-Bahía Inglesa. Rev geológica Chile. 2007;34: 81–96. 10.4067/S0716-02082007000100005

[17] Avise JC, Arnold J, Ball RM, Bermingham E, Lamb T, Neigel JE, et al Intraspecific Phylogeography: The Mitochondrial DNA Bridge Between Population Genetics and Systematics. Annu Rev Ecol Syst. 1987;18: 489–522. 10.1146/annurev.es.18.110187.002421

[18] Avise JC. Phylogeography: retrospect and prospect. J Biogeogr. 2009;36: 3–15. 10.1111/j.1365-2699.2008.02032.x

[19] Excoffier L, Smouse PE, Quattro JM. Analysis of molecular variance inferred from metric distances among DNA haplotypes: application to human mitochondrial DNA restriction data. Genetics. 1992;131: 479–91. 1644282 10.1093/genetics/131.2.479 PMC1205020

[20] Campos-Soto R, Torres-Pérez F, Solari A. Phylogenetic incongruence inferred with two mitochondrial genes in *Mepraia* spp. and *Triatoma eratyrusiformis* (Hemiptera, Reduviidae). Genet Mol Biol. 2015;38: 390–395. 10.1590/S1415-475738320140301 26500444 PMC4612603

[21] WHISPers [Internet]. [cited 1 May 2020]. Available: https://whispers.usgs.gov/home

[22] Folmer O, Black M, Hoeh W, Lutz R, Vrijenhoek R. DNA primers for amplification of mitochondrial cytochrome c oxidase subunit I from diverse metazoan invertebrates. Mol Mar Biol Biotechnol. 1994;3: 294–299. 10.1371/journal.pone.0013102 7881515

[23] Monteiro FA, Barrett T V, Fitzpatrick S, Cordon-Rosales C, Feliciangeli D, Beard CB. Molecular phylogeography of the Amazonian Chagas disease vectors *Rhodnius prolixus* and *R*. *robustus*. Mol Ecol. 2003;12: 997–1006. 1802 [pii] 10.1046/j.1365-294x.2003.01802.x 12753218

[24] Hall TA. BioEdit: a user-friendly biological sequence alignment editor and analysis program for Windows 95/98/NT. Nucleic Acids Symp. 1999;41: 95–98. citeulike-article-id:691774

[25] Thompson JD, Higgins DG, Gibson TJ. CLUSTAL W: improving the sensitivity of progressive multiple sequence alignment through sequence weighting, position-specific gap penalties and weight matrix choice. Nucleic Acids Res. 1994;22: 4673–4680. 10.1093/nar/22.22.4673 7984417 PMC308517

[26] Rozas J, Ferrer-Mata A, Sanchez-DelBarrio JC, Guirao-Rico S, Librado P, Ramos-Onsins SE, et al DnaSP 6: DNA sequence polymorphism analysis of large data sets. Mol Biol Evol. 2017;34: 3299–3302. 10.1093/molbev/msx248 29029172

[27] Lefort V, Longueville JE, Gascuel O. SMS: Smart Model Selection in PhyML. Mol Biol Evol. 2017;34: 2422–2424. 10.1093/molbev/msx149 28472384 PMC5850602

[28] Guindon S, Gascuel O. A simple, fast, and accurate algorithm to estimate large phylogenies by maximum likelihood. Syst Biol. 2003;52: 696–704. 10.1080/10635150390235520 14530136

[29] Bouckaert R, Heled J, Kühnert D, Vaughan T, Wu CH, Xie D, et al BEAST 2: A Software Platform for Bayesian Evolutionary Analysis. PLoS Comput Biol. 2014;10: e1003537 10.1371/journal.pcbi.1003537 24722319 PMC3985171

[30] Baele G, Lemey P, Bedford T, Rambaut A, Suchard MA, Alekseyenko A V. Improving the accuracy of demographic and molecular clock model comparison while accommodating phylogenetic uncertainty. Mol Biol Evol. 2012;29: 1157–1167. 10.1093/molbev/mss084 22403239 PMC3424409

[31] Rambaut A, Suchard M, Xie W, Drummond AJ. Tracer v. 1.6. Institute of Evolutionary Biology, University of Edinburgh. Available from http://beast.bio.ed.ac.uk/Tracer; 2014.

[32] Arensburger P, Buckley T. Biogeography and phylogeny of the New Zealand cicada genera (Hemiptera: Cicadidae) based on nuclear and mitochondrial DNA data. J Biogeogr. 2004;31: 557–569. 10.1046/j.1365-2699.2003.01012.x

[33] Bandelt HJ, Forster P, Röhl A. Median-joining networks for inferring intraspecific phylogenies. Mol Biol Evol. 1999;16: 37–48. 10.1093/oxfordjournals.molbev.a026036 10331250

[34] Leigh JW, Bryant D. PopART: full-feature software for haplotype network construction. Nakagawa S, editor. Methods Ecol Evol. 2015;6: 1110–1116. 10.1111/2041-210X.12410

[35] Beerli P. Comparison of Bayesian and maximum-likelihood inference of population genetic parameters. Bioinformatics. 2006;22: 341–345. 10.1093/bioinformatics/bti803 16317072

[36] Beerli P. How to use MIGRATE or why are Markov chain monte Carlo programs difficult to use? In: Giorgio B, Bruford M, Hauffe H, Rizzoli A, Vernesi C, editors. Population Genetics for Animal Conservation. Cambridge, UK: Cambridge University Press; 2009 pp. 42–79. 10.1017/CBO9780511626920.004

[37] Wickham H. ggplot2: Elegant Graphics for Data Analysis. Second Edition Springer. Media 2016 10.1007/978-0-387-98141-3

[38] Excoffier L, Lischer HEL. Arlequin suite ver 3.5: A new series of programs to perform population genetics analyses under Linux and Windows. Mol Ecol Resour. 2010;10: 564–567. 10.1111/j.1755-0998.2010.02847.x 21565059

[39] Karesh WB, Dobson A, Lloyd-Smith JO, Lubroth J, Dixon MA, Bennett M, et al Ecology of zoonoses: Natural and unnatural histories. Lancet. 2012;380: 1936–1945. 10.1016/S0140-6736(12)61678-X 23200502 PMC7138068

[40] Canals M, Cruzat L, Molina MC, Ferreira A, Cattan PE. Blood host sources of *Mepraia* spinolai (Heteroptera: Reduviidae), wild vector of Chagas Disease in Chile. J Med Entomol. 2001;38: 303–307. 10.1603/0022-2585-38.2.303 11296839

[41] Canals M, Ehrenfeld M, Cattan PE. Situation of *Mepraia spinolai*, a wild vector for Chagas disease in Chile, compared to others vectors, from the perspective of their alimentary profile. Rev Med Chil. 2000;128: 1108–1112. 10.4067/S0034-98872000001000005 11349509

[42] Forattini OP, Rocha e Silva EO, Ferreira OA, Rabello EX, Pattoli DG. Aspectos ecológicos da tripanossomose americana. 3. Dispersão local de triatomíneos, com especial referência ao *Triatoma sordida*. Rev Saude Publica. 1971; 10.1590/S0034-89101971000200002 5003316

[43] Gourbière S, Dorn P, Tripet F, Dumonteil E. Genetics and evolution of triatomines: From phylogeny to vector control. Heredity (Edinb). 2012;108: 190–202. 10.1038/hdy.2011.71 21897436 PMC3282395

[44] Abrahan LB, Gorla DE, Catalá SS. Dispersal of *Triatoma infestans* and other triatominae speciesin the arid Chaco of Argentina—Flying, walking or passive carriage? Mem Inst Oswaldo Cruz. 2011;106: 232–239. 10.1590/s0074-02762011000200019 21537686

[45] Cohen KM, Finney SC, Gibbard PL, Fan J-X. The ICS International Chronostratigraphic Chart. Episodes, J Int Geosci. 2013;36: 199–204. 10.18814/epiiugs/2013/v36i3/002

[46] Ceccarelli FS, Pizarro-Araya J, Ojanguren-Affilastro AA. Phylogeography and population structure of two *Brachistosternus* species (Scorpiones: Bothriuridae) from the Chilean coastal desert—the perils of coastal living. Biol J Linn Soc. 2017;120: 75–89. 10.1111/bij.12877

[47] Campos R, Botto-Mahan C, Coronado X, Catala SS, Solari A. Phylogenetic relationships of the spinolai complex and other Triatomini based on mitochondrial DNA sequences (Hemiptera: Reduviidae). Vector borne zoonotic Dis. 2013;13: 73–6. 10.1089/vbz.2011.0954 23199269

[48] Moreno ML, Gorla D, Catalá S. Association between antennal phenotype, wing polymorphism and sex in the genus *Mepraia* (Reduviidae: Triatominae). Infect Genet Evol. 2006;6: 228–234. 10.1016/j.meegid.2005.06.001 16081323

[49] Calleros L, Panzera F, Bargues MD, Monteiro FA, Klisiowicz DR, Zuriaga MA, et al Systematics of *Mepraia* (Hemiptera-Reduviidae): Cytogenetic and molecular variation. Infect Genet Evol. 2010;10: 221–228. 10.1016/j.meegid.2009.12.002 20018255

[50] Campos-Soto R, Panzera F, Pita S, Lages C, Solari A, Botto-Mahan C. Experimental crosses between *Mepraia gajardoi* and *M*. *spinolai* and hybrid chromosome analyses reveal the occurrence of several isolation mechanisms. Infect Genet Evol. 2016;45: 205–212. 10.1016/j.meegid.2016.09.001 27600593

[51] Kierszenbaum F, Ivanyi J, Budzko DB. Mechanisms of natural resistance to trypanosomal infection. Role of complement in avian resistance to *Trypanosoma cruzi* infection. Immunology. 1976;30: 1–6. 765264 PMC1444967

[52] Urdaneta-Morales S, McLure I. Experimental infections in Venezuelan lizards by *Trypanosoma cruzi*. Acta Trop. 1981;38: 99–105. 10.5169/seals-312810 6115559

