# Supplementary material for: Biogeographic origin and phylogenetic relationships of Mepraia (Hemiptera, Reduviidae) on islands of northern Chile
Source: PLoS One. 2020 Jun 11;15(6):e0234056. doi: 10.1371/journal.pone.0234056 (PMC7289421; doi:10.1371/journal.pone.0234056)
Supplement: S1 Data — (DOCX) [file pone.0234056.s001.docx]

**Haplotypes per species and GenBank Accession numbers**

|  |  |  |  | **GenBank accession number** | |
| --- | --- | --- | --- | --- | --- |
| **Haplotype** | **Species** | **N** | **Locality** | ***COI*** | ***cyt b*** |
| Outgroup | *T. eratyrusiformis* | 1 | La Rioja* | MN117859 | KC236979 |
| Outgroup | *T. breyeri* | 1 | La Rioja* | MN117860 | KC236980 |
| H1 | *M. gajardoi* | 8 | Corazones* | KC236913 | KC236946 |
| H2 | *M. gajardoi* | 2 | Corazones* | KC236914 | KC236947 |
| H3 | *M. gajardoi* | 1 | Corazones* | KC236915 | KC236948 |
| H4 | *M. gajardoi* | 1 | Corazones* | KC236916 | KC236949 |
| H5 | *M. gajardoi* | 1 | Corazones* | KC236917 | KC236950 |
| H6 | *M. gajardoi* | 2 | Caleta Vitor* | KC236918 | KC236951 |
| H7 | *M. gajardoi* | 3 | Caleta Vitor* | KC236919 | KC236952 |
| H8 | *M. gajardoi* | 7 | Caleta Vitor * | KC236920 | KC236953 |
| H9 | *M. gajardoi* | 7 | Caleta Vitor, Camarones * | KC236921 | KC236954 |
| H10 | *M. gajardoi* | 7 | Caleta Vitor * | KC236922 | KC236955 |
| H11 | *M. gajardoi* | 9 | Río Seco* | KC236923 | KC236956 |
| H12 | *M. gajardoi* | 6 | Río Seco, San Marcos* | KC236924 | KC236957 |
| H13 | *M. gajardoi* | 2 | Río Seco * | KC236925 | KC236958 |
| H14 | *M. gajardoi* | 3 | Río Seco * | KC236926 | KC236959 |
| H15 | *M. gajardoi* | 6 | San Marcos * | KC236927 | KC236960 |
| H16 | Undetermined | 12 | SM-I | MN117861 | MN117875 |
| H17 | Undetermined | 4 | SM-I | MN117862 | MN117876 |
| H18 | Undetermined | 12 | SM-C | MN117863 | MN117877 |
| H19 | Undetermined | 5 | SM-C | MN117864 | MN117878 |
| H20 | *M. parapatrica* | 62 | 25 PA-I, 37 PA-C | MN117865 | MN117879 |
| H21 | *M. parapatrica* | 4 | PA-I | MN117866 | MN117880 |
| H22 | *M. parapatrica* | 2 | PA-I | MN117867 | MN117881 |
| H23 | *M. parapatrica* | 2 | PA-I | MN117868 | MN117882 |
| H24 | *M. parapatrica* | 2 | PA-I | MN117869 | MN117883 |
| H25 | *M. parapatrica* | 5 | PA-C | MN117870 | MN117884 |
| H26 | *M. parapatrica* | 11 | Medano* | KC236928 | KC236961 |
| H27 | *M. parapatrica* | 5 | Caleta Zenteno* | KC236929 | KC236962 |
| H28 | *M. parapatrica* | 11 | Caleta Zenteno* | KC236930 | KC236963 |
| H29 | *M. spinolai* | 1 | Inca | MN117871 | MN117885 |
| H30 | *M. spinolai* | 7 | Inca | MN117872 | MN117886 |
| H31 | *M. spinolai* | 1 | Inca | MN117873 | MN117887 |
| H32 | *M. spinolai* | 2 | Llanos de Challe* | KC236931 | KC236964 |
| H33 | *M. spinolai* | 1 | Llanos de Challe * | KC236932 | KC236965 |
| H34 | *M. spinolai* | 2 | Llanos de Challe * | KC236933 | KC236966 |
| H35 | *M. spinolai* | 3 | Llanos de Challe * | KC236934 | KC236967 |
| H36 | *M. spinolai* | 13 | Alto del Carmen* | KC236935 | KC236968 |
| H37 | *M. spinolai* | 1 | Alto del Carmen * | KC236936 | KC236969 |
| H38 | *M. spinolai* | 14 | Caleta Toro* | KC236937 | KC236970 |
| H39 | *M. spinolai* | 5 | Montepatria* | KC236938 | KC236971 |
| H40 | *M. spinolai* | 8 | Montepatria * | KC236939 | KC236972 |
| H41 | *M. spinolai* | 1 | Montepatria * | KC236940 | KC236973 |
| H42 | *M. spinolai* | 5 | Illapel RNCh* | KC236941 | KC236974 |
| H43 | *M. spinolai* | 2 | Illapel RNCh * | KC236942 | KC236975 |
| H44 | *M. spinolai* | 4 | Illapel RNCh * | KC236943 | KC236976 |
| H45 | *M. spinolai* | 4 | SF | MN117874 | MN117888 |
| H46 | *M. spinolai* | 2 | Til Til* | KC236944 | KC236977 |
| H47 | *M. spinolai* | 9 | Til Til * | KC236945 | KC236978 |

N: Number of individuals; *: Latitude/longitude available in Campos et al. (2013) [7]; Latitude/longitude of localities of this study are available in Table 1.

**Details of sequence editing and alignment**

The sequences were edited separately for each gene. The forward and reverse sequences of each sample were inspected separately to confirm the correct nucleotide. Only samples that showed clear peaks in the chromatogram were included in the analyses. Reverse sequences were aligned with forward sequences using the reverse complement tool to reveal potential reading inconsistencies, and only congruent nucleotides were used in the analyses. Samples sequences with noisy chromatograms were not included in the analysis. Then, we performed the alignment with the previously published sequences available in the GenBank for each gene. Alignments were performed with the Clustal W tool implemented in the BioEdit software, using "Full Multiple alignment" and bootstrap NJ tree with 1000 iterations (default parameters). After the alignments, sites that showed nucleotide substitutions were re-examined by visual inspection of each individual’s raw chromatogram. Some sites were excluded (using the delete tool) to obtain sequences of the same length of those sequences downloaded from GenBank, resulting a final matrix without gaps. The sequences of the *COI* gene (508 pb) of each sample were manually concatenated with their respective *cyt b* gene sequence (514 pb) obtaining a final matrix of 1022 bp.
